# Impact of acetylsalicylic acid in patients undergoing cerebral aneurysm surgery – should the neurosurgeon really worry about it?

**DOI:** 10.1007/s10143-021-01476-7

**Published:** 2021-01-25

**Authors:** Ali Rashidi, Nadine Lilla, Martin Skalej, I. Erol Sandalcioglu, Michael Luchtmann

**Affiliations:** 1 Department of Neurosurgery, Otto-von-Guericke University Magdeburg, Leipziger Str. 44, Magdeburg, Germany; 2 Department of Neuroradiology, Otto-von-Guericke University Magdeburg, Leipziger Str. 44, Magdeburg, Germany

**Keywords:** Acetylsalicylic acid, Aneurysm surgery, Clipping, Postoperative hemorrhage

## Abstract

There has been an increase in the use of acetylsalicylic acid (ASA, Aspirin®) among patients with stroke and heart disease as well as in aging populations as a means of primary prevention. The potentially life-threatening consequences of a postoperative hemorrhagic complication after neurosurgical operative procedures are well known. In the present study, we evaluate the risk of continued ASA use as it relates to postoperative hemorrhage and cardiopulmonary complications in patients undergoing cerebral aneurysm surgery. We retrospectively analyzed 200 consecutive clipping procedures performed between 2008 and 2018. Two different statistical models were applied. The first model consisted of two groups: (1) group with *No ASA impact* - patients who either did not use ASA at all as well as those who had stopped their use of the ASA medication in time (> = 7 days prior to operation); (2) group with *ASA impact* - all patients whose ASA use was not stopped in time. The second model consisted of three groups: (1) *No ASA use*; (2) *Stopped ASA use* (> = 7 days prior to operation); (3) *Continued ASA use* (did not stop or did not stop in time, <7 days prior to operation). Data collection included demographic information, surgical parameters, aneurysm characteristics, and all hemorrhagic/thromboembolic complications. A postoperative hemorrhage was defined as relevant if a consecutive operation for hematoma removal was necessary. An ASA effect has been assumed in 32 out of 200 performed operations. A postoperative hemorrhage occurred in one out these 32 patients (3.1%). A postoperative hemorrhage in patients without ASA impact was detected and treated in 5 out of 168 patients (3.0%). The difference was statistically not significant in either model (*ASA impact group* vs. *No ASA impact group*: OR = 1.0516 [0.1187; 9.3132], *p* = 1.000; RR = 1.0015 [0.9360; 1.0716]). Cardiopulmonary complications were significantly more frequent in the group with ASA impact than in the group without ASA impact (*p* = 0.030). In this study continued ASA use was not associated with an increased risk of a postoperative hemorrhage. However, cardiopulmonary complications were significantly more frequent in the *ASA impact* group than in the *No ASA impact* group. Thus, ASA might relatively safely be continued in patients with increased cardiovascular risk and cases of emergency cerebrovascular surgery.

## Introduction

Low-dose Acetylsalicylic acid (ASA) is recommended as preventive treatment for many disorders, including coronary artery diseases (CAD) and stroke, as well as for patients receiving a percutaneous coronary or carotid artery stenting to prevent stent-thrombosis. In addition, many patients suffering from atrial fibrillation use aspirin or other anticoagulant therapies for prevention of stroke [5, 6, 8, 14, 18, 21, 27, 30, 37, 42, 43, 46, 48, 52].

By transfer of its acetyl group to serine residue in the platelet cyclooxygenase receptor ASA irreversibly inhibits platelet function. It prevents the configuration of thromboxane A2 and subsequenty the aggregation of platelet for the entire lifecycle (7–10 days) [19, 20, 28, 31, 35, 41]. The inhibition of platelet function ensues even at lower dosages [10, 11, 44]. Abrupt interruption of ASA use may lead to a hypercoagulability, which are associated with some of the major adverse cardiovascular events that have been reported [33].

ASA has been proved as a major risk factor in the development of postoperative hemorrhage following neurosurgical procedures [36, 40]. Because postoperative rebleeding is frequently a life-threatening complication after intracranial operations, ASA is usually discontinued seven days or more prior to elective intracranial procedures [22, 28].

Weighting the risks of perioperative hemorrhage and cardiovascular complications of patients using ASA is a challenging task in daily neurosurgical routine. Yet, only a very limited number of studies evaluated the risk of ASA discontinuation or continuation before and after the neurosurgical treatment. There are a limited number of studies that have shown worse outcomes with pre-injury antiplatelet treatment [12, 16, 23, 24, 49]. However, a clear consensus on how to handle patients using ASA in the perioperative settings does not yet established [13, 17, 20, 25, 26, 32, 38, 47].

In the present study, we have evaluated the perioperative ASA management as it relates to the risk of postoperative hemorrhage and adverse cardiovascular events in patients undergoing neurosurgical treatment of ruptured and unruptured intracranial aneurysms.

## Methods

This retrospective study analyzed the medical records of all consecutive patients who underwent clipping procedures at our institution between 2008 and 2018. All aneurysms were treated via “traditional” microsurgical clipping. In only one patient two aneurysm were sealed in one session. In all other SAH patients, the ruptured aneurysm only was clipped.

Clinical data on patients were obtained by retrospective chart review, and included

- age
- sex
- blood group
- body mass index (BMI)
- perioperative use of ASA
- hypertension
- smoking history
- laboratory parameters
- subarachnoid hemorrhage/unruptured aneurysm
- length of hospitalization
- Hunt and Hess scale
- Fisher Grade
- location and size of aneurysm
- multiplicity of aneurysms
- operative procedure
- op-duration
- blood loss
- complications (hemorrhage, deep vein thrombosis, etc.)
- peri- and postoperative Karnofsky performance status (KPS), Glasgow outcome score (GOS)

In 43 patients the data records revealed ASA in the recent medical history. Of these, 11 patients discontinued the ASA use timely prior to surgery. However, in 32 patients the ASA use was not stopped at all or timely. The common ASA dosage was 100 mg, except in three patients, were the ASA dosage was 250 mg, 500 mg and 1000 mg.

To test for possible ASA impact on postoperative hemorrhage two different statistical models utilizing Fisher’s exact test were applied. The first model consisted of two groups:

- *No ASA impact -* patients who either had not used ASA medication at all or who had stopped the use of ASA at or before the recommended time (> = 7 days prior to operation)
- *ASA impact* - all patients who had not discontinued ASA use within the recommended time frame

The second model consisted of three groups:

- *No ASA use*
- *Stopped ASA use* (> = 7 days prior to operation);
- *Continued ASA use* (did not stop use <7 days prior to operation or did not stop use at all).

A postoperative hemorrhage was defined as relevant if a subsequent operation for hematoma removal was necessary. Major thromboembolic complications were defined as myocardial infarction and/or pulmonary embolism.

Data storage and statistical analyses were performed using the software package SAS University Edition (SAS Institute, Inc., Cary, New York, USA). Because the present analysis was performed in an explorative sense, it was deliberately reviewed to the full level of significance. Each *p* value <0.05 therefore represented a statistically significant result. For unadjusted analyses, Fisher’s exact test was applied for categorical variables, and the robust t test (Satterthwaite) was used for continuous variables, where variables with extreme deviation from normal distribution had been log transformed.

## Results

In total, 200 clipping operations were performed between 2008 and 2018. In the current study, we considered the most important complication after clipping surgery and divided it into two categories: postoperative hemorrhagic events and thromboembolic events. We studied potential risk factors of hemorrhagic complications regardless of ASA status. We evaluated the effects of demographic characteristics, aneurysm characteristics, hospitalization, and surgical features.

**Fig. 1:**
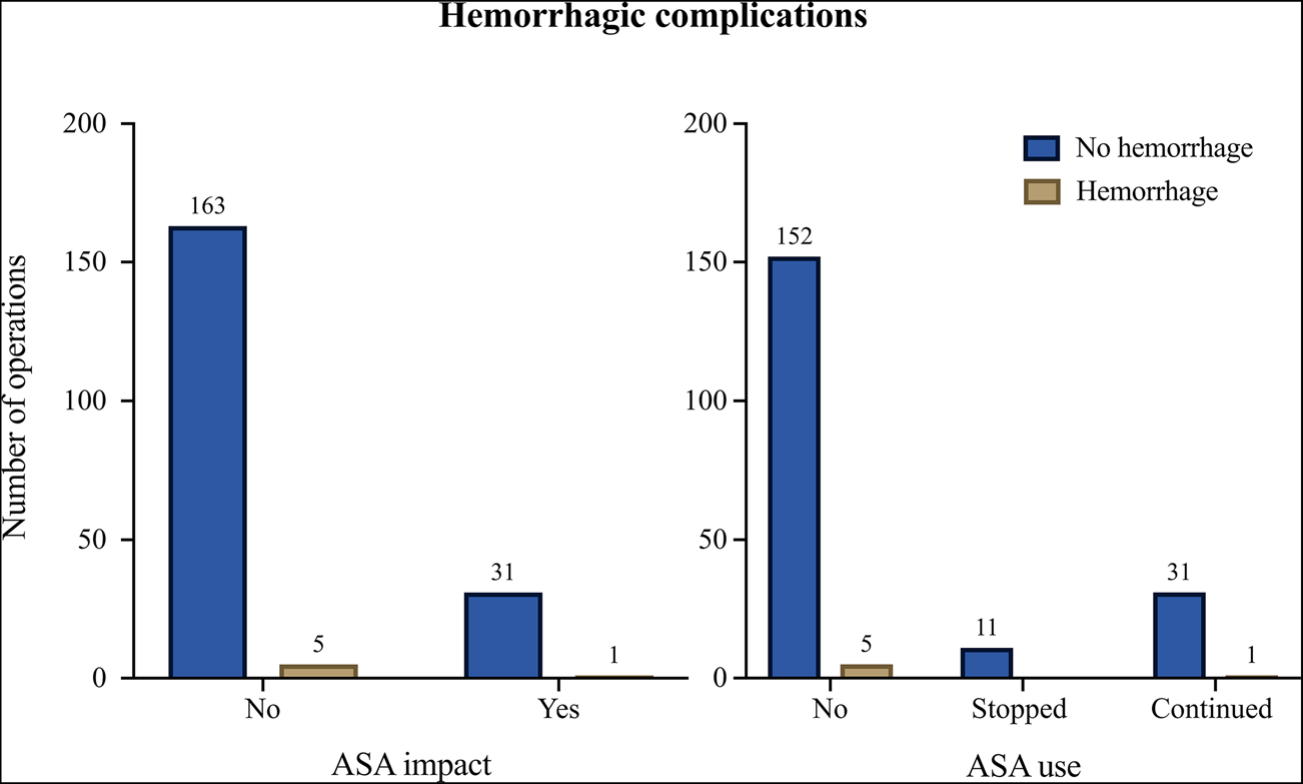
- Number of hemorrhagic complications per ASA condition. No significant difference in the risk of postoperative hemorrhage between the three conditions was obtained (*p* = 0.835)

**Fig. 2:**
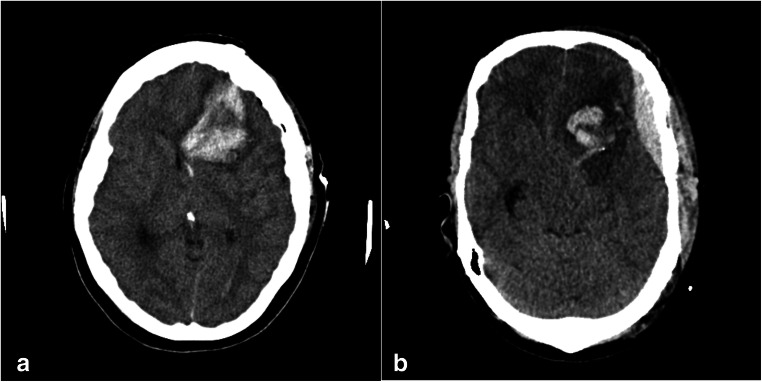
- Two representative cases of postoperative hemorrhage after clipping of middle cerebral artery aneurysms: (A) intracerebral hemorrhage, (B) epidural hematoma

Postoperative hemorrhagic and thromboembolic complications were observed in less than 3% of the operations. Figure 2 shows two representative cases of postoperative hemorrhage. A total of 6 patients suffered a postoperative hemorrhage. Of these, one patient had used ASA (Table 1). Table 2 shows that there is no significant difference in postoperative hemorrhage occurrence in the different groups. Re-craniotomy with evacuation of a hematoma was required no more frequently in the *ASA impact* group than in the *No ASA impact* group (*p* = 1.0). As also shown in Fig. 1 and Table 2, in the case of ASA use, there is also no significant difference in the risk of postoperative hemorrhage between the three conditions (*p* = 0.835).

**Table 1:** – Demographic and aneurysm characteristics of patients suffered from postoperative hemorrhage

					**Hemorrhage**		**Aneurysm**		
**#**	**Age [years]**	**Gender**	**SAH**	**ASA**	Type	Side	Location	Side	Size [mm]
1	78	female	yes	100 mg	intracerebral	ipsilateral	MCA	right	3.4
2	57	female	no	no	intracerebral	contralateral	MCA	right	3.3
3	65	female	yes	no	subdural	ipsilateral	MCA	left	4
4	62	female	no	no	subgaleal	ipsilateral	MCA	left	9
5	45	male	yes	no	epidural	ipsilateral	MCA	right	3.6
6	63	female	yes	no	intracerebral	ipsilateral	MCA	left	5

**Table 2:** – No evidence for increased risk from continued ASA use has been found

				**Hemorrhage**		
		**ASA dosage**	**∑**	**No**	**Yes**	
		mean ± SD [mg]	N (% column)	N (% row)	N (% row)	**p value**
**ASA impact**	**No**	6.5 ± 24.8	168 (84.0)	163 (97.0)	5 (3.0)	1.000
	**Yes**	145.3 ± 172.9	32 (16.0)	31 (96.9)	1 (3.1)	
**ASA use**	**No**	0.0 ± 0.0	157 (78.5)	152 (96.8)	5 (3.2)	0.835
	**Stopped**	100.0 ± 0.0	11 (5.5)	11 (100.0)	0 (0.0)	
	**Continued**	145.3 ± 172.9	32 (16.0)	31 (96.9)	1 (3.1)	
	**∑**		200	194 (97.0)	6 (3.0)

Age and sex of patients and other demographic characteristics such as body mass index (BMI), blood group, hypertension and smoking history were nearly equally distributed. There was no significant difference in patient age between the *No ASA impact* group (*N* = 168 / 55.6 ± 11.4 years) and the *ASA impact* group (*N* = 32 / 59.4 ± 14.0 years) [*p* = 0.150]. These demographic characteristics are presented in Table 3 and do not impact on the postoperative hemorrhagic frequency in the different groups.

**Table 3:** – Demographic characteristics of the investigated patients

	**Operations**	**No ASA impact**	**ASA impact**	**p value**	**No hemorrhage**	**Hemorrhage**	**p value**
	N (%)	N (%) / mean ± SD	N (%) / mean ± SD		N (%) / mean ± SD	N (%) / mean ± SD	
**Demographic information**							
Age [years]	200	168 / 55.6 ± 11.4	32 / 59.4 ± 14.0	0.150	194 / 56.0 ± 11.9	6 / 61.7 ± 10.8	0.259
Gender	200						
female	139 (69.5)	117 (84.2)	22 (15.8)	1.000	134 (96.4)	5 (3.6)	0.669
male	61 (30.5)	51 (83.6)	10 (16.4)		60 (98.4)	1 (1.6)	
Height [cm]	187	156 / 169.1 ± 8.1	31 / 168.0 ± 9.9	0.574	182 / 168.9 ± 8.0	5 / 170.2 ± 18.7	0.884
Weight (kg)	187	156 / 74.2 ± 14.1	31 / 75.5 ± 14.0	0.638	182 / 74.3 ± 14.1	5 / 77.4 ± 14.6	0.665
Body mass index	187	156 / 25.9 ± 4.6	31 / 26.7 ± 4.5	0.355	182 / 26.0 ± 4.6	5 / 26.9 ± 4.5	0.690
Blood group (AB0)	180						
A	71 (39.4)	60 (84.5)	11 (15.5)	0.483	71 (100.0)	0 (0.0)	0.100
B	25 (13.9)	23 (92.0)	2 (8.0)		24 (96.0)	1 (4.0)	
AB	9 (5.0)	8 (88.9)	1 (11.1)		8 (88.9)	1 (11.1)	
0	75 (41.7)	59 (78.7)	16 (21.3)		72 (96.0)	3 (4.0)	
Blood group (rhesus)	180						
Rh+	155 (86.1)	126 (81.3)	29 (18.7)	0.083	151 (97.4)	4 (2.6)	0.531
Rh-	25 (13.9)	24 (96.0)	1 (4.0)		24 (96.0)	1 (4.0)	
Hypertension	200						
Yes	89 (44.5)	72 (80.9)	17 (19.1)	0.334	87 (97.8)	2 (2.2)	0.694
No	111 (55.5)	96 (86.5)	15 (13.5)		107 (96.4)	4 (3.6)	
Smoker	200						
Yes	70 (35.0)	58 (82.9)	12 (17.1)	0.840	68 (97.1)	2 (2.9)	1.000
No	130 (65.0)	110 (84.6)	20 (15.4)		126 (96.9)	4 (3.1)	

Clipping was performed in 148 cases on patients who suffered subarachnoid hemorrhage (SAH) and in 52 cases on patients who had unruptured intracranial aneurysms (UIA). Out of the 32 patients operated under the impact of ASA 24 suffered from SAH. Subsequent urgent operations prevented the timely withdrawal of ASA. In the remaining 8 patients, discontinuation of ASA would have resulted in an increased cardiovascular risk (e.g. following recent placement of coronary stents). There was no significant difference in distribution between the *No ASA impact* (*N* = 124 SAH vs. *N* = 44 UIA) and the *ASA impact* groups (*N* = 24 SAH vs. *N* = 8 UIA) [*p* = 1,000]. The classification according to the Hunt and Hess scale, the modified Fisher grade in patients with SAH, and the length of hospitalization in both groups, as well as any thrombosis prophylaxis, showed no significant difference between the *No ASA impact* and the *ASA impact* groups. However, there was a significant difference in length of hospitalization (Table 4) between patients without postoperative hemorrhage (*N* = 194 / 15.6 ± 9.1 days) and patients with postoperative hemorrhage (*N* = 6 / 25.0 ± 8.7 days) (*p* = 0.045).

**Table 4:** – Clinical parameters. Unsurprisingly, patients with postoperative hemorrhaging spent a significantly longer time in hospital

	**Operations**	**No ASA impact**	**ASA impact**	**p value**	**No hemorrhage**	**Hemorrhage**	**p value**
	N (%)	N (%) / mean ± SD	N (%) / mean ± SD		N (%) / mean ± SD	N (%) / mean ± SD	
**Hospitalization/SAH**							
Duration [days]	200	168 / 15.9 ± 9.5	32 / 16.2 ± 7.7	0.845	194 / 15.6 ± 9.1	6 / 25.0 ± 8.7	**0.045**
Subarachnoid hemorrhage	200						
Yes	148 (74.0)	124 (83.8)	24 (16.2)	1.000	144 (97.3)	4 (2.7)	0.651
UIA / No	52 (26.0)	44 (84.6)	8 (15.4)		50 (96.1)	2 (3.9)	
Hunt and Hess scale	138						
1	42 (30.4)	35 (83.3)	7 (16.7)	0.407	40 (95.2)	2 (4.8)	0.296
2	24 (17.4)	22 (91.7)	2 (8.3)		22 (91.7)	2 (8.3)	
3	17 (12.3)	12 (70.6)	5 (29.4)		17 (100.0)	0 (0.0)	
4	43 (31.2)	35 (81.4)	8 (18.6)		43 (100)	0 (0.0)	
5	12 (8.7)	11 (91.7)	5 (8.3)		12 (100.0)	0 (0.0)	
Fisher grade (modified)	137						
0	2 (1.5)	2 (100.0)	0 (0.0)	0.649	2 (100.0)	0 (0.0)	0.305
1	4 (3.0)	4 (100.0)	0 (0.0)		4 (100.0)	0 (0.0)	
2	21 (15.3)	18 (85.7)	3 (14.3)		20 (95.2)	1 (4.8)	
3	53 (38.7)	46 (86.8)	7 (13.2)		50 (94.3)	3 (5.7)	
4	57 (41.6)	44 (77.2)	13 (22.8)		57 (100.0)	0 (0.0)	
Thromboprophylaxis	188						
No	176 (93.6)	149 (84.7)	27 (15.3)	0.411	170 (96.6)	6 (3.4)	1.000
Yes	12 (6.4)	9 (75.0)	3 (25.0)		12 (100.0)	0 (0.0)	

With regard to a postoperative hemorrhage, aneurysm size proved to play a role and was the only statistically significant parameter (N = 194: 7.1 ± 4.6 mm vs. N = 6: 4.7 ± 2.2; *p* = 0.042). As shown in Table 5, all other analyzed aneurysm characteristics did not significantly correlate with the risk of postoperative hemorrhage.

**Table 5:** – Aneurysm characteristics. The size of the aneurysm might be a factor influencing the risk of postoperative hemorrhage

	**Operations**	**No ASA impact**	**ASA impact**	**p value**	**No hemorrhage**	**Hemorrhage**	**p value**
	N (%)	N (%) / mean ± SD	N (%) / mean ± SD		N (%) / mean ± SD	N (%) / mean ± SD	
**Aneurysm characteristics**							
Aneurysm location	200						
MCA	134 (67.0)	109 (81.3)	25 (18.7)	0.155	128 (95.5)	6 (4.5)	0.874
Acom	31 (15.5)	29 (93.6)	2 (6.4)		31 (100.0)	0 (0.0)	
ACA	16 (8.0)	15 (93.7)	1 (6.3)		16 (100.0)	0 (0.0)	
ACI	12 (6.0)	10 (83.3)	2 (16.7)		12 (100.0)	0 (0.0)	
PICA	3 (1.5)	1 (33.3)	2 (66.7)		3 (100.0)	0 (0.0)	
Pcom	2 (1.0)	2 (100.0)	0 (0.0)		2 (100.0)	0 (0.0)	
VA	2 (1.0)	2 (100.0)	0 (0.0)		2 (100.0)	0 (0.0)	
Circulation	200						
Anterior	195 (97.5)	165 (84.6)	30 (15.4)	0.182	189 (96.9)	6 (3.1)	1.000
Posterior	5 (2.5)	3 (60.0)	2 (40.0)		5 (100.0)	0 (0.0)	
Hemisphere	200						
Right	115 (57.5)	97 (84.3)	18 (15.7)	1.000	112 (97.4)	3 (2.6)	0.788
Left	85 (42.5)	71 (83.5)	14 (16.5)		82 (96.5)	3 (3.5)	
Size [mm]	200	168 / 7.1 ± 4.6	32 / 7.0 ± 4.0	0.992	194 (7.1 ± 4.6)	6 (4.7 ± 2.2)	**0.042**
Number of aneurysms							
Single	128 (64.0)	110 (85.9)	18 (14.1)	0.323	122 (95.3)	6 (4.7)	0.089
Multiple	72 (36.0)	58 (80.6)	14 (19.4)		72 (100.0)	0 (0.0)	
Shape of aneurysm	200						
Saccular	144 (72.0)	118 (81.9)	26 (18.1)	0.283	142 (98.6)	2 (1.4)	0.053
Multilobar	56 (28.0)	50 (89.3)	6 (10.7)		52 (92.9)	4 (7.1)	

Blood loss during surgery was similar for the *No ASA impact* group (197.6 ± 46.5 ml) and the *ASA-impact* group (203.9 ± 40.5 ml; *p* = 0.438). The other operative parameters (Table 6), such as duration of surgery and application of temporary clipping did not play a role in the occurrence of a postoperative hemorrhage. The perioperative laboratory parameters including international normalized ratio (INR), partial thromboplastin time (PTT) [sec], thrombin time (TT) [sec] and platelet count (10^9^/l) showed no significant difference between the study groups.

**Table 6:** – Perioperative parameters have no significant influence on the risk of postoperative hemorrhaging

		**Operations**	**No ASA impact**	**ASA impact**	**p value**	**No hemorrhage**	**Hemorrhage**	**p value**
		N (%)	N (%) / mean ± SD	N (%) / mean ± SD		N (%) / mean ± SD	N (%) / mean ± SD	
**Operative parameters**								
Operation time [min]		200	168 / 197.6 ± 46.5	32 / 203.9 ± 40.5	0.438	194 / 198.6 ± 46.0	6 / 197.7 ± 27.0	0.938
Temporary clipping (sec)		48	37 / 143.2 ± 125.6	11 / 245.4 ± 165.6	0.080	46 / 164.5 ± 141.8	2 / 215.5 ± 142.1	0.701
Blood loss (ml)		185	154 / 365.2 ± 341	31 / 348.5 ± 220.5	0.730	180 / 364.7 ± 327.0	5 / 282.6 ± 138.9	0.270
**Laboratory parameters**								
International normalized ratio (INR)		173	144 / 1.0 ± 0.1	29 / 1.0 ± 0.1	0.852	168 / 1.0 ± 0.1	5 / 1.0 ± 0.1	0.945
Partial thromboplastin time (PTT) [sec]		200	168 / 28.0 ± 2.4	32 / 27.5 ± 3.0	0.383	194 / 27.9 ± 2.5	6 / 28.0 ± 2.4	1.000
Thrombin time (TT) [sec]		192	161 / 16.0 ± 2.0	31 / 15.9 ± 2.1	0.823	186 / 16.0 ± 2.1	6 / 16.1 ± 0.7	0.800
Platelet count (10^9^/l)		200	168 / 261.8 ± 85.3	32 / 280.4 ± 90.7	0.289	194 / 266.0 ± 87.0	6 / 225.8 ± 45.7	0.148
**Performance status/Outcome**								
KPS postoperative		199	168 / 72.3 ± 28.0	31 / 64.8 ± 3.2	0.245	193 / 71.5 ± 29.0	6 / 60.0 ± 26.1	0.334
Glasgow outcome score (GOS)		199	168 / 4.2 ± 1.1	31 / 3.7 ± 1.4	0.095	193 / 4.1 ± 1.2	6 / 4.0 ± 1.3	0.843

Table 7 shows the frequency of cardiopulmonary complications of the investigated patients. Five patients suffered a pulmonary embolism after a deep vein thrombosis. One of these patients had not taken ASA. In one patient the ASA medication was discontinued in time. Three patients suffered pulmonary embolism under ASA impact. This type of complication was significantly more frequent in the *ASA impact* group than in the *No ASA impact* group (*p* = 0.030). However, the occurrence of cardiopulmonary complications was independent of whether ASA use had been discontinued or not been used at all prior to surgery.

**Table 7:** – Cardiopulmonary complications were significantly more frequent in the *ASA-impact* group than in the *No-ASA-impact* group. However, a short-term discontinuation of ASA treatment seems to have had no influence on the incidence of perioperative thromboembolic complications

			**Cardiopulmonary complications**		
		**∑**	**No**	**Yes**	
		N (% column)	N (% row)	N (% row)	**p value**
**ASA impact**	**No**	168 (84.0)	166 (98.8)	2 (1.2)	**0.030**
	**Yes**	32 (16.0)	29 (90.6)	3 (9.4)	
**ASA use**	**No**	157 (78.5)	156 (99.4)	1 (0.6)	**0.009**
	**Stopped**	11 (5.5)	10 (90.9)	1 (9.1)	
	**Continued**	32 (16.0)	29 (90.6)	3 (9.4)	
	**∑**	200	194 (97.0)	6 (3.0)	
**ASA use**	**Continued**	32 (74.4)	29 (90.6)	3 (9.4)	1.000
	**Stopped**	11 (25.6)	10 (90.9)	1 (9.1)	
	**∑**	43	39 (90.7)	4 (9.3)	

## Discussion

We investigated the impact of perioperative ASA treatment on postoperative hemorrhage, thromboembolic complications and clinical parameters in patients undergoing aneurysm-clipping operations for ruptured and unruptured intracranial aneurysms. In our study cohort, we could not observe that perioperative ASA use does result in increased hemorrhagic complications for aneurysm-clipping surgery.

In some studies, aneurysm characteristics such as size and posterior circulation location, as well as demographic characteristics such as patient age and case volume were seen as risk factors for surgical treatment of UIA [1–3, 34, 51, 53]. In our study, aneurysm size proved to be an independent risk factor indicating that smaller aneurysms are associated with an increased risk of postoperative hemorrhage. This observation is contrary to the findings by Nakamizo et al. [38], who did not observe an impact of aneurysms size on the rate of postoperative hemorrhage.

Nakamizo et al. [38] reveal in their study on patients undergoing craniotomy for UIA that intracranial hemorrhage was more frequent in the group that was receiving antithrombotic treatment than in the group not receiving antithrombotic drugs. In a study by Toyoda et al. [50] it was found that neither double treatment with antiplatelet inhibitors nor the addition of an antiplatelet inhibitor to warfarin placed patients at increased risk of perioperative bleeding. An aggravated postoperative hemorrhage risk due to the intake of ASA could not be confirmed in our study. Hanalioglu et al. [20] demonstrated - similar to our study - that perioperative ASA use was not associated with an increased rate of hemorrhagic complications after intracranial tumor surgery.Unsurprisingly, we found a significant difference in length of hospitalization time between those patients with postoperative hemorrhage compared to patients without hemorrhage. It is relatable that for patients with postoperative hemorrhage, the recovery phase and thus the length of hospital stay is longer.

In our study, cardiopulmonary complications were significantly more frequent in the *ASA impact* group than in the *No ASA impact* group. Some studies suggest that discontinuing ASA medication in patients receiving antithrombotic treatment can pose a significant risk factor of both thromboembolic and bleeding events [4, 15]. The international, randomized, placebo-controlled trial (POISE-2) revealed that the perioperative use of ASA had no significant impact on mortality, but increased the risk of major hemorrhage [9]. Our results are somehow conflicting with the assumption of Gerstein et al. [15] who supposed that abrupt discontinuation of long-term ASA usage could lead to platelet rebound phenomena resulting in increased risk of thrombosis. The role of ASA (−withdraw) in the context of deep venous thrombosis and pulmonary embolism after SAH remains unclear. During the study period, postoperative thrombosis prophylaxis using low molecular weight heparin was not administrated routinely for cranial surgery, thus, based on our data reliable conclusions cannot be drawn [45]. For the prevention of venous thromboembolism most patients used compression stockings only.

Recent guidelines in anesthesiology and cardiology recommend perioperative ASA continuation in patients with low to moderate bleeding risk and high cardiovascular risk [7, 39]. Intracranial operations as well as intramedullary spinal surgeries are considered high-risk bleeding procedures and are therefore excluded from recommendations for ASA continuation, even in patients with a high cardiovascular risk [39]. A national survey of neurosurgeons in Germany studying the use of ASA prior to intracranial operations revealed that 77.5% of those responding believed that patients taking low-dose ASA had an increased risk of major perioperative hemorrhage [28]. Of those respondents, 58% reported personal experience with perioperative bleeding. The same group also reported the results of a study on spinal surgery that found a similar percentage of perioperative bleeding occurrence. Nevertheless, in a related study only a small fraction (5%) of neurosurgeons would agree to perform spinal surgery under ASA medication [29].

However, some limiting aspects beyond the obvious flaws of retrospective study designs have to be discussed. In the reported period functional platelet testing was not available in time for most patients. Therefore, statistical analysis was not feasible and reliable conclusions regarding ASA non-responder were not possible. Furthermore, the treatment strategies were different in several cases, particularly in patients with multiple aneurysms. However, due to the relatively low incidence of posthemorrhagic events subgroup analysis were not useful.

## Conclusion

In this study continued ASA use was not associated with an increased risk of a postoperative hemorrhage in patients undergoing aneurysm surgery. In contrast, cardiopulmonary complications were significantly more frequent in the *ASA-impact* group than in the *No-ASA-impact* group. However, in patients with increased cardiovascular risk and in the case of emergency surgery, ASA might relatively safe be continued. Aneurysms size was found to be an independent risk factor regarding the incidence of postoperative hemorrhage. Prospective randomized studies are necessary to evaluate the risks and safety of the perioperative use of ASA in neurosurgical procedures with respect on epidemiological changes and increased use of antiplatelet treatment or anticoagulation.

## Data Availability

Not applicable.
